# A systematic review and meta-analysis of randomised controlled trials in the management of neovascular glaucoma: absence of consensus and variability in practice

**DOI:** 10.1007/s00417-022-05785-5

**Published:** 2022-08-08

**Authors:** Saajan Ramji, Gurnoor Nagi, Abdus Samad Ansari, Obeda Kailani

**Affiliations:** 1 The Medical School, Guy’s King’s & St Thomas’ Medical School, King’s College London, University of London, London, UK; 2 Department of Ophthalmology, King’s College Hospital NHS Foundation Trust, Queen Mary’s Hospital, London, UK; 3 Section of Academic Ophthalmology, School of Life Course Sciences, FoLSM, King’s College London, London, UK

**Keywords:** Neovascular glaucoma, Randomised controlled trial, Anti-VEGF, Surgical, Systematic review, Meta-analysis

## Abstract

**Purpose:**

Neovascular glaucoma (NVG) is characterised by neovascularisation of the angle and therefore elevated intraocular pressure (IOP). This results in progressive optic neuropathy and loss of visual acuity. Treatment aims to reduce IOP in order to prevent optic nerve damage. A systematic review was completed synthesising results from randomised control trials (RCTs) comparing interventions for the management of NVG and their efficacy and safety.

**Methods:**

Data was sourced from Web of Science, Embase and Medline after 1st January 2000. The primary outcome measures were mean IOP at follow-up and success rate. The secondary outcomes included mean IOP lowering medications and total complications. A meta-analysis was completed on comparative studies using Revman (version 5.4).

**Results:**

For the two studies comparing Ahmed glaucoma valve (AGV) + pan-retinal photocoagulation (PRP) vs AGV + PRP + intra-vitreal bevacizumab (IVB), there was no difference in mean IOP or odds of success from the meta-analysis. From the 4 studies examining the utilisation of anti-vascular endothelial growth factor (anti-VEGF), one study showed lower mean IOP at 1 (*p* = 0.002) and 3 months (*p* = 0.033) for IVB vs sham injection. In the 2 studies studying transcleral diode laser (TDL), there were no significant findings. From the 4 studies looking at trabeculectomy (trab), lower mean IOP at 6 (*p* = 0.001), 9 (*p* = 0.01), 12 (*p* = 0.02) and 18 months (*p* = 0.004) was shown for intra-vitreal ranibizumab (IVR) + PRP + visco-trabeculectomy vs IVR + PRP + trab, and a significantly lower mean IOP was present in the Baerveldt group vs trab at 6 months (*p* = 0.03). In the 2 studies investigating the AGV, there was a lower mean IOP at 1 month (*p* = 0.01) in the AGV + triamcinolone (TCA) group. The risk of bias was low for 4 studies, high for 4 studies and 6 studies had some concerns.

**Conclusion:**

This is the first meta-analysis of RCTs in the management of neovascular glaucoma. The lack of high-quality evidence contributes to the lack of consensus in managing NVG. Our results highlight modern treatment strategies and the need for better powered RCTs with long-term follow-up in order to establish optimal treatment modalities and true patient outcomes.

**Supplementary Information:**

The online version contains supplementary material available at 10.1007/s00417-022-05785-5.

## Introduction


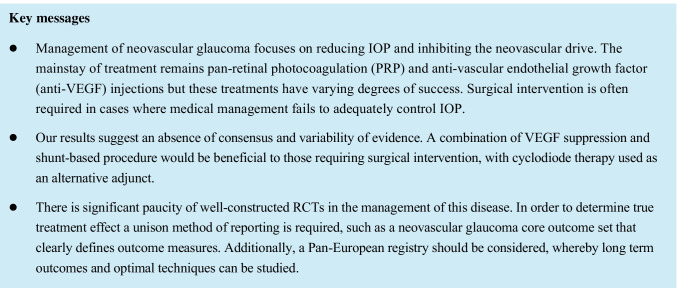


Neovascular glaucoma (NVG) is a form of secondary glaucoma characterised by elevated intraocular pressure (IOP) and neovascularisation of the angle, culminating in damage to the optic nerve. It arises secondary to posterior segment ischaemia. Causes include central retinal vein occlusion (CRVO), central retinal artery obstruction (CRAO), proliferative diabetic retinopathy (DR) and ocular ischaemic syndrome (OIS) [1]. Initial neovascularisation is due to stimulation of angiogenesis secondary to retinal ischaemia [2]. This results in the production of vascular endothelial growth factor (VEGF) and neovascularisation of the iris [3]. This obstructs the iris and anterior chamber iridocorneal angle, eventually leading to angle-closure glaucoma with raised IOP [3–5].

The disease often leads to complete loss of vision and therefore early diagnosis and management is essential.

Clinically, NVG has the following features: neovascularisation of the iris (NVI), neovascularisation of the angle (NVA) and raised IOP. On slit-lamp examination NVI is visible as thin vessels, tortuous in nature, arranged in a non-organised pattern. These vessels are found on the iris surface and near the pupillary margin [1]. Corneal oedema may be present depending on degree of IOP increase. On gonioscopy, the iridocorneal angle can be open initially; as the disease progresses, the angle develops neovascularisation; this ultimately leads to complete angle closure [2].

Management of NVG focuses on reducing IOP and inhibiting the neovascular drive. Initially, this is in the form of topical and systemic ocular antihypertensive medications; however, the mainstay of treatment remains pan-retinal photocoagulation (PRP) and anti-VEGF injections to manage retinal ischaemia and neovascularisation [6]. These treatments have varying degrees of success depending on factors such as aetiology and severity of disease.

Surgical intervention is often required in cases where medical management fails to adequately control IOP to prevent optic nerve damage. These surgical techniques aim to either decrease inflow with continuous or pulsed laser cyclophotocoagulation or enhance drainage with a fistula [7]. Glaucoma drainage devices such as the Ahmed glaucoma valve, Paul’s tube and Preserflo microshunt have been utilised in the management of raised intraocular pressure in the context of NVG [8]. However, NVG in itself is a risk factor for failure of these devices particularly, in subjects presenting with poor visual acuities preoperatively or postoperative raised IOP [9]. The neovascular drive similarly impacts success rates for trabeculectomy (Trab); however, the use of antimetabolites such as mitomycin C, as an adjunct, has led to improved rates of success [10].

Delayed presentation and diagnosis of NVG is an issue due to it being a late presentation of the primary systemic or ocular disorder [11] and has been further exacerbated by the COVID-19 pandemic [12]. Deferring medical attention in patients with underlying retinal ischaemia may lead not only to increased incidence, but increased severity of NVG at diagnosis. This systematic review will aim to analyse outcomes and efficacy of treatment reported in randomised controlled trials (RCTs) for the management of NVG.

## Methods

### Search strategy

**Fig1:**
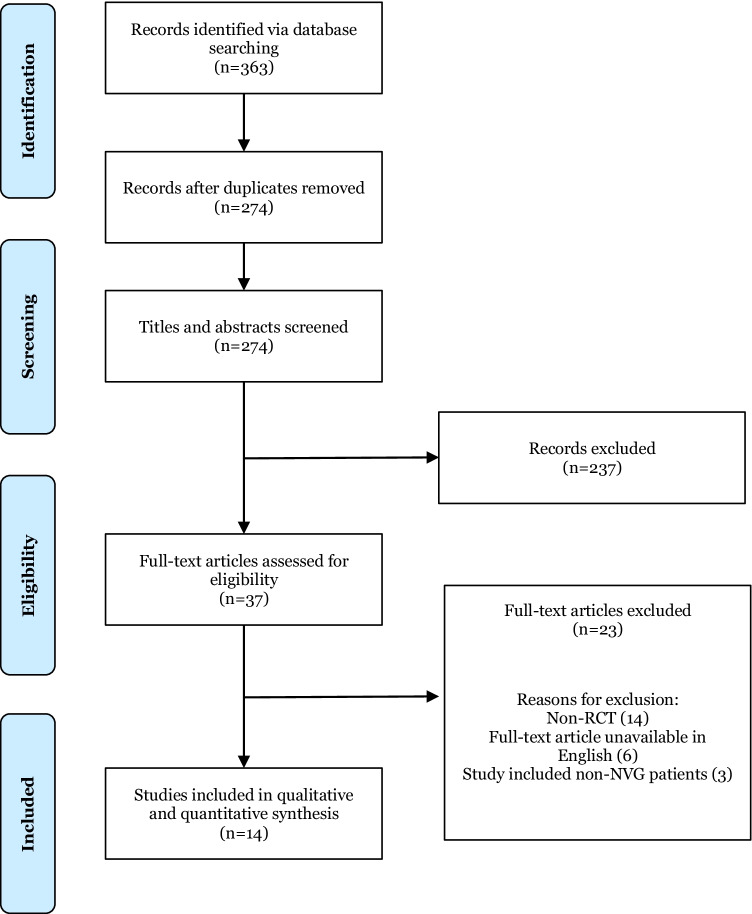
PRISMA flowchart of studies identified, screened and included

This review was registered on PROSPERO, ID: CRD42021298021. Web of Science, Embase and Medline were all systematically searched for studies published between 1st January 2000 and 31st December 2021 (Appendix A) and were reported according to the Preferred Reporting Items for Systematic Reviews and Meta-analyses (PRISMA) criteria (Fig. 1). Duplicates were removed before the articles were screened through a two-stage process by two independent authors (SR, GN). Initially, both authors, SR and GN, screened all articles from the search separately by title and secondly by abstract using Rayyan software [13]. Disputes at this stage regarding inclusion and exclusion were discussed between both authors and settled by consensus. Next, the full articles were read and evaluated against the eligibility criteria. Disputes regarding inclusion and exclusion of articles were again discussed between all authors and settled by consensus. In instances where consensus could not be reached, advice was sought from a third reviewer (ASA). Reference lists of all included studies were also screened to identify additional studies.

### Data extraction

Data from included studies was extracted into a data collection table. Information extracted from the studies included the study design, study population demographics (inclusion criteria), sample size and population characteristics (age, gender and ethnicity), the definition and measure for the outcome as well as results relevant to our review, which included mean IOP, mean number of IOP lowering medications, complications and success rates. In addition, we contacted corresponding authors of studies to request additional data from analysis referred to in the methodology, but not reported in the manuscript.

Data extraction was completed by 2 authors (SR and GN) independently for all 14 RCTs and was assessed for accuracy and completion to ensure all relevant data was accurately captured. Any discrepancies were discussed, and a third reviewer (ASA) was consulted when consensus could not be established.

### Eligible studies

All RCTs that compared treatments for neovascular glaucoma were included. Non-English studies were excluded unless there was an English translation available at the time of search. Studies involving living human participants comprising and between the dates 1st January 2000 and 31st December 2021 were included to reflect modern practice. Studies involving non-neovascular phenotypes of glaucoma were excluded, as were in vitro and non-human studies.

### Eligible participants

Eligible participants were male or female adult patients, over the age of 18, with neovascular glaucoma of any underlying aetiology undergoing treatment with any intervention for neovascular glaucoma for IOP lowering intent.

### Eligible interventions and comparators

The intervention and comparators comprised of a variety of treatments and procedures aimed to treat neovascular glaucoma. These included shunts such as the Ahmed glaucoma valve (AGV), high-pressure AGV (High AGV), express implant and the Baerdveldt implant. There were also laser treatments such as transcleral diode laser (TDL) and pan-retinal photocoagulation (PRP), as well as anti-VEGF treatments: intra-vitreal afilibercept (IVA), intra-vitreal ranibizumab (IVR) and intra-vitreal bevacizumab (IVB). Studies also investigated combination of surgical, medical and laser treatments: high-pressure AGV (high AGV) + partial Tenon’s capsule resection (PTCR), AGV + mitomycin C (MMC), AGV + 5-fluoruracil (5-FU), AGV + PRP, AGV + PRP + IVB, AGV + triamcinolone (TCA), PRP + IVB, PRP + IVR + viscotrabeculectomy (Vtrab), PRP + IVR + Trab, Trab + 1.25 mg intra-cameral bevacizumab (ICB), Trab + 2.5 mg ICB, Trab + PRP + intra-vitreal conbercept (IVC) and Trab + PRP + IVR and trabeculectomy alone (Table 1).

**Table 1:** Baseline characteristics of all included studies

Study ID (author, country and year of publication)	Control vs comparator		Number of patients			Gender		Mean age (years)		Predisposing diagnosis (number, %)		Mean follow-up duration (months)	
			**Total** **(eyes)**	**Control** **(eyes)** **(R/L)**	**Comparator** **(eyes)** **(R/L)**	**Control** **(M/F)**	**Comparator** **(M/F)**	**Control** **(mean ± SD)**	**Comparator** **(mean ± SD)**	**Control**	**Comparator**	**Control** **(mean ± SD)**	**Comparator** **(mean ± SD)**
AGV + PRP vs AGV + PRP + IVB													
Mahdy et al., Egypt (2013)	AGV + PRP	AGV + PRP + IVB	40 (40)	20 (20) (NA)	20 (20) (NA)	11/9	12/8	56 ± 4.3	53 ± 1.1	DR 15 (75) CRVO 4 (20) OIS 1 (5)	DR 16 (80) CRVO 4 (20)	18	18
Arcieri et al., Brazil (2015)	AGV + PRP	AGV + PRP + IVB	40 (40)	20 (20) (14/6)	20 (20) (9/11)	11/9	13/7	62.40 ± 11.78	59.25 ± 8.05	DR 11 (55) CRVO 9 (45)	DR 10 (50) CRVO (50)	24	24
Utilisation of anti-VEGF													
Yazdani et al., Iran (2009)	Sham injection	IVB	26 (26)	12 (12) (NA)	14 (14) (NA)	9/3	12/2	62.4 ± 12.7	57.6 ± 16.9	DR 7 (58.3) CRVO 5 (41.6) Uveitis 0 (0)	DR 9 (64.3) CRVO 4 (28.6) Uveitis 1 (7.1)	5.8 ± 1.5	6.1 ± 1.1
Wittstrom et al., Sweden (2012)	PRP	PRP + IVB	19 (19)	9 (9) (NA)	10 (10) (NA)	5/4	2/8	78.0 ± 8.7	78.4 ± 7.8	NA	NA	6	6
Guo et al., China 2021	Trab + PRP + IVC	Trab + PRP + IVR	160 (160)	80 (80) (NA)	80 (80) (NA)	48/32	44/36	62	64	DR 60 (75) CRVO 18 (22.5) OIS 2 (2.5)	DR 54 (67.5) CRVO 21 (26.25) OIS 5 (6.25)	12	12
Inatani et al., Japan (2021)	Sham injection	IVA	52 (52)	27 (27) (NA)	27 (27) (NA)	22/5	23/4	66.2 ± 14	68.1 ± 13	DR 13 (48) CRVO 5 (19) OIS 5 (19) Other 4 (15)	DR 10 (37) CRVO 11 (41) OIS 4 (15) Other 2 (7)	3.25	3.25
Transcleral diode laser (TDL)													
Wagdy et al., Egypt (2020)	Express	TDL	28 (30)	12 (12) (NA)	16 (18) (NA)	6/6	6/10	48.66 ± 4.81	46.25 ± 5.49	DR 8 (66.7) CRVO 4 (33.3)	DR 11 (61.1) CRVO 7 (39.9)	12	12
Choy et al., China (2018)	TDL	AGV	21 (22)	8 (9) (3/6)	13 (13) (4/9)	5/1	8/5	61.3 ± 13.5	62.8 ± 11.0	DR 4 CRVO 1 OIS 3	DR 6 CRVO 4 CRAO 1 OIS 1	28.5 ± 17.9	31.0 ± 15.4
Trabeculectomy (Trab)													
Sisto et al., Italy (2007)	Trab + MMC	Trab + 5-FU	40 (40)	22 (22) (NA)	18 (18) (NA)	14/8	12/6	59.7 ± 9.8	63.5 ± 9.4	DR 14 (63.6) CRVO 4 (18.1) Idiopathic 4 (18.1)	DR 10 (55.5) CRVO 6 (33.3) Idiopathic 2 (11.1)	18.6 ± 17.2	35.8 ± 22.6
Gupta et al., India (2009)	Trab + ICB 1.25	Trab + ICB 2.5	19 (19)	9 (9) (NA)	10 (10) (NA)	4/5	6/4	51.1	57.7	DR 3 (33.3) CRVO 4 (44.4) Uveitis 2 (22.2)	DR 4 (40) CRVO 5 (50) Eales disease 1 (10)	6	6
Elwehidy et al., Egypt (2019)	IVR + PRP + Trab	IVR + PRP + Vtrab	51 (51)	25 (25) (12/13)	26 (26) (15/11)	14/11	14/12	52.4 ± 8.8	54.1 ± 6.4	DR 20 (80) CRVO 5 (20)	DR (21 (80.8) CRVO 5 (19.2)	18	18
Tokumo et al., Japan (2021)	Baerveldt	Trab	50 (50)	23 (23) (NA)	27 (27) (NA)	13/10	15/12	66 ± 13.7	62.6 ± 10.9	DR 18 (78.2) CRVO 1 (4.3) BRVO 3 (13) OIS 1 (4.3) Unknown 0	DR 23 (85.2) CRVO 3 (11.1) BRVO 0 OIS 0 Unknown 1 (3.7)	26 ± 19.4	27.3 ± 20.1
Ahmed glaucoma valve (AGV)													
Susanna Jr et al., Brazil (2003)	AGV	High AGV + PTCR	92 (92)	47 (47) (NA)	45 (45) (NA)	25/22	25/20	60.7 ± 12.2	60.6 ± 12.1	DR 30 CRVO 17	DR 28 CRVO 17	12	12
Teixeria et al., Brazil (2012)	AGV	AGV + TCA	49 (49)	27 (27) (NA)	22 (22) (NA)	15/12	16/6	57.48 ± 15.32	62.91 ± 7.26	DR 15 (55.6) CRVO 12 (44.4)	DR 9 (40.9) CRVO 13 (59.1)	12	12

*R/L*, right/left; *M/F*, male/female; *SD*, standard deviation; *DR*, diabetic retinopathy; *CRVO*, central retinal vein occlusion; *OIS*, ocular ischaemic syndrome; *BRVO*, branched retinal vein occlusion; *AGV*, Ahmed glaucoma valve; *High AGV*, high-pressure AGV; *PTCR*, partial Tenon’s capsule resection; *Trab*, trabeculectomy; *Vtrab*, visco-trabeculectomy; *MMC*, mitomycin C; *5-FU*, 5-flurouracil; *IVB*, intra-vitreal bevacizumab; *IVR*, intra-vitreal ranibizumab; *IVA*, intra-vitreal afilibercept; *ICB*, intra-cameral bevacizumab; *PRP*, pan-retinal photocoagulation; *TCA*, triamcinolone; *TDL*, transcleral diode laser

### Outcome measures

The primary outcome measures were mean IOP at 1 month, 6 months, 12 months, 24 months and at the final follow-up as well as success rates at final follow-up. Secondary outcomes were mean IOP lowering medications at 1 month, 6 months, 12 months, 24 months and at the final follow-up as well as the total number of complications throughout the entire follow-up period. The definition for success rates for studies varied and can be found in Table 2.

**Table 2:** Success rate and complications

Study ID	Interventions		Number of patients and eyes			Definition of success	Success rate % (success/total)			Complications (total)	
	Control	Comparator	Total (eyes)	Control (eyes) (R/L)	Comparator (eyes) (R/L)		Control	Comparator	*p* value	Control	Comparator
AGV + PRP vs AGV + PRP + IVB											
Mahdy et al., Egypt (2013)	AGV + PRP	AGV + PRP + IVB	40 (40)	20 (20) (NA)	20 (20) (NA)	IOP < 21 and > 10 mmHg with no IOP lowering medication or further surgery (complete success) Above with IOP lowering medications (Qualified)	Complete 20 (5/25) Qualified 20 (5/25)	Complete 75 (15/20) Qualified 20 (4/20)	Complete *p* < 0.05	41	14
Arcieri et al., Brazil (2015)	AGV + PRP	AGV + PRP + IVB	40 (40)	20 (20) (14/6)	20 (20) (9/11)	Success criteria 1: IOP < 21 mmHg and with or without IOP lowering medications Success criteria 2: IOP reduction of > 30% compared to pre-operative values	Success criteria 1 60 12/20 Success criteria 2 75 (15/20)	Success criteria 1 65 13/20 Success criteria 2 80 (16/20)	Criteria 1 *p* = 0.2838 Criteria 2 *p* = 0.3012	15	9
Utilisation of anti-VEGF											
Yazdani et al., Iran (2009)	Sham injection	IVB	26 (26)	12 (12)	14 (14)	-	-	-	-	-	-
Wittstrom et al., Sweden (2012)	PRP	PRP + IVB	19 (19)	9 (9) (NA)	10 (10) (NA)	-	-	-	-	-	-
Guo et al., China (2021)	Trab + PRP + IVC	Trab + PRP + IVR	160 (160)	80 (80) (NA)	80 (80) (NA)	IOP between 8 and 18 mmHg at 1-year follow-up	-	-	*p* = 0.536	15	20
Inatani et al., Japan (2021)	Sham	IVA	52 (52)	27 (27) (NA)	27 (27) (NA)	-	-	-	-	20	13
Transcleral diode laser (TDL)											
Choy et al., China (2018)	TDL	AGV	21 (22)	8 (9) (3/6)	13 (13) (4/9)	IOP < 21 mmHg with or without IOP lowering medication and preserved or improved BCVA	63 (5/8)	42 (5/12)	*p* = 0.65	-	11
Wagdy et al., Egypt (2020)	Express	TDL	28 (30)	12 (12) (NA)	16 (18) (NA)	IOP < 22 mmHg and no further treatment (complete) Above but with medical treatment (qualified)	Complete 50 (6/12) Qualified 41.7 (5/12)	Complete 44.44 (8/18) Qualified 38.9 (7/16)	-	6	5
Trabeculectomy (Trab)											
Sisto et al., Italy 2007	Trab + MMC	Trab + 5-FU	40 (40)	22 (22)	18 (18)	IOP < 21 mmHg with topical treatment (qualified) or without topical treatment (complete)	54.5 (12/22) Qualified 45.4 (10/22) Complete 9.1 (2/22)	55.5 (10/18) Qualified 11.1 (2/18) Complete 44.4 (8/18)	-	14	22
Gupta et al., India (2009)	Trab + ICB 1.25	Trab + ICB 2.5	19 (19)	9 (9)	10 (10)	-	-	-	-	5	8
Elwehidy et al., Egypt (2019)	IVR + PRP + Trab	IVR + PRP + Vtrab	51 (51)	25 (25) (12/13)	26 (26) (15/11)	IOP < 21 and > 6 mmHg with no further surgical procedures and no IOP lowering medications (complete) Above criteria but with IOP lowering medications (qualified)	Complete 52 (13/25) Qualified 28 (7/25)	Complete 53.8 (14/26) Qualified 30.8 (8/26)	Qualified *p* = 0.726	49	42
Tokumo et al., Japan (2021)	Baerveldt	Trab	50 (50)	23 (23) (NA)	27 (27) (NA)	IOP < 22 mmHg or > 20% reduction in IOP with no further surgeries and no decrease in BCVA	59.1 (14/23)	61.6 (16/27)	*p* = 0.71	31	23
Ahmed glaucoma valve (AGV)											
Susanna Jr et al., Brazil (2003)	AGV	High AGV + PTCR	92 (92)	47 (47)	45 (45)	IOP > 4 and < 22 mmHg and at least 30% reduction of an IOP reduction ± IOP lowering medication	77.7 (NA)	70.4 (NA)	*p* > 0.05	28	29
Teixeria et al., Brazil (2012)	AGV	AGV + TCA	49 (49)	27 (27) (NA)	22 (22) (NA)	IOP < 21 and > 6 mmHg (success) using < 2 IOP lowering medications (complete success)	Success 76 (NA) Complete success 64 (NA)	Success 77.8 (NA) Complete success 77.8 (NA)	Success *p* = 0.82 Complete *p* = 0.31	26	23

*R/L*, right/left; *AGV*, Ahmed glaucoma valve; *High AGV*, high-pressure AGV; *PTCR*, partial Tenon’s capsule resection; *Trab*, trabeculectomy; *Vtrab*, visco-trabeculectomy; *MMC*, mitomycin C; *5-FU*, 5-flurouracil; *IVB*, intra-vitreal bevacizumab; *IVR*, intra-vitreal ranibizumab; *IVA*, intra-vitreal afilibercept; *ICB*, intra-cameral bevacizumab; *PRP*, pan-retinal photocoagulation; *TCA*, triamcinolone; *TDL*, transcleral diode laser

^*^p-value is given for success rates between control and comparator but percentage success is not stated. It is only represented on a Kaplan–Meier curve; no figures are stated

### Assessment of risk of bias and quality of effect

The risk of bias assessment was carried out with the Cochrane Risk of Bias 2.0 tool for randomised-controlled trials [14]. The quality of our effect estimates was assessed using the GRADE rating system [15].

### Data analysis

The interventions and comparators were compared via a narrative synthesis. Continuous variables (mean IOP and mean IOP lowering medications) were measured by the mean with standard deviation; categorical variables (success rate) were measured by percentages and have been presented in tables and figures.

A quantitative meta-analysis using Review Manager (RevMan [Computer Program]. Version 5.4, The Cochrane Collaboration, 2020) was carried out for 2 studies that compared the same interventions [16, 17] to compare mean IOP and success rates. For the studies included in the meta-analysis, complete success, as defined in both studies, respectively, was used as the success rate. Studies that contained data with disparate outcomes were not included in the meta-analysis; instead, these were discussed in the narrative synthesis.

## Results

### Search results

Overall, 14 RCTs were included in this systematic review and meta-analysis. The initial search yielded a total of 363 studies. After removal of duplicates, 274 articles were screened by title and abstract against the inclusion criteria. This resulted in 37 articles that underwent full text screening. From these, 14 were included in this systematic review for both quantitative and qualitative analysis. In accordance with the Preferred Reporting Items for Systematic Reviews and Meta-Analyses (PRISMA) guidelines, a flow diagram for the results of the study selection procedure is shown in Fig. 1.

### Study characteristics

Search criteria included all studies meeting the inclusion criteria, between the date ranges of 1st January 2000 and 31st December 2021. This resulted in a total of 687 patients and 690 eyes undergoing various surgical and medical interventions for NVG. The mean age for all patients was 60.5 ± 7.3 years of age and the mean follow-up was 15.2 ± 9 months (Table 1).

Two studies compared AGV + PRP with AGV + PRP + IVB [16, 17]. Four studies looked at the addition of anti-VEGF. One study was by Wittstrom et al. (2012) which compared PRP with PRP + IVB and another 2 studies [18], one by Inatani et al. (2021) and another by Yazdani et al. (2009), compared sham injection with IVA or IVB, respectively [19, 20]. Guo et al. (2021) compared Trab + PRP + IVC vs Trab + PRP + IVR [21].

Two RCTs investigated TDL against other surgical interventions, Express Implant [22] and AGV [23]. Tokumo et al. (2021) compared Trab vs the Baerveldt shunt [24] and Elwehidy et al. (2019) compared IVR + PRP + Trab with IVR + PRP + visco-trabeculectomy (Vtrab) [25]. Two more studies investigated trabeculectomy treatment; Sisto et al. (2007) compared Trab + MMC with Trab + 5-FU [26] and Gupta et al., (2009) compared Trab with either 1.25 mg of ICB vs 2.5 mg ICB [27]. Finally, Teixeria et al. (2012) compared AGV with AGV + TCA [28] and Susanna Jr et al. (2003) compared AGV with high-pressure AGV (High AGV) + Partial Tenon’s capsule resection (PTCR) [29] (Table 1).

### Risk of bias

All studies were evaluated for risk of bias by two independent authors (SR and GN) with the Cochrane ROB 2.0 tool [30]. Any disputes were settled following a discussion. Four studies had a low risk of bias [16, 19, 20, 27]. From the remaining 10 studies, 6 had some concerns [17, 18, 21, 23, 26, 28] and 4 had a high risk of bias [22, 24, 25, 29] (Table 3).

**Table 3:** Risk of bias for randomised comparative studies using the RoB (Risk of Bias) 2.0 tool

Study ID (author, country and year of publication)	Bias from randomisation	Bias from effect of assignment to intervention	Bias from effect of effect of adhering to intervention	Bias due to missing outcome data	Bias in measurement of outcome	Bias in selection of reported result	Overall risk of bias
AGV + PRP vs AGV + PRP + IVB							
Mahdy et al., Egypt (2013)	Low risk	Low risk	Low risk	Low risk	Low risk	Low risk	Low
Arcieri et al., Brazil (2015)	Low risk	Some concerns	Low risk	Low risk	Low risk	Low risk	Some concerns
Utilisation of anti-VEGF							
Yazdani et al., Iran (2009)	Low risk	Low risk	Low risk	Low risk	Low risk	Low risk	Low
Wittstrom et al., Sweden (2012)	Some concerns	Low risk	Low risk	Low risk	Low risk	Low risk	Some concerns
Guo et al., China (2021)	Low risk	Some concerns	Low risk	Low risk	Low risk	Low risk	Some concerns
Inatani et al., Japan (2021)	Low risk	Low risk	Low risk	Low risk	Low risk	Low risk	Low
Transcleral diode laser (TDL)							
Wagdy et al., Egypt (2020)	Some concerns	Some concerns	Low risk	Low risk	Low risk	Low risk	High
Choy et al., China (2018)	Some concerns	Low risk	Low risk	Low risk	Low risk	Low risk	Some concerns
Trabeculectomy (Trab)							
Sisto et al., Italy (2007)	Low risk	Low risk	Low risk	Low risk	Low risk	Some concerns	Some concerns
Gupta et al., India (2009)	Low risk	Low risk	Low risk	Low risk	Low risk	Low risk	Low
Elwehidy et al., Egypt (2019)	Some concerns	Some concerns	Low risk	Low risk	Low risk	Low risk	High
Tokumo et al., Japan (2021)	High risk	Low risk	Low risk	Low risk	Some concerns	Low risk	High
Ahmed glaucoma valve (AGV)							
Susanna Jr et al., Brazil (2003)	High risk	Some concerns	Low risk	Low risk	Low risk	Some concerns	High
Teixeria et al., Brazil (2012)	Low risk	Some concerns	Low risk	Low risk	Low risk	Low risk	Some concerns

## AGV + PRP vs AGV + PRP + IVB

### Success rates

**Fig. 2:**
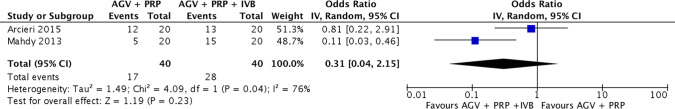
Meta-analysis of complete success rates at final follow-up. AGV, Ahmed glaucoma valve; PRP, pan-retinal photocoagulation; IVB, intra-vitreal bevacizumab; SD, standard deviation; CI, confidence interval

Complete success at the final follow-up was used for meta-analysis. No statistical difference was observed between the 2 interventions (OR = 0.31, 95% CI [0.04, 2.15] *p* = 0.23) (Fig. 2). The success rates for one study were defined as IOP < 21 mmHg and > 10 mmHg with no further IOP lowering medication or surgery as complete success, and with IOP lowering medications as qualified success [16]. For the second study, IOP < 21 mmHg with or without IOP lowering medications was deemed one criteria of success and this was used in the meta-analysis. The other criteria for success were IOP reduction of > 30% from baseline [17].

### Mean IOP

**Fig. 3:**
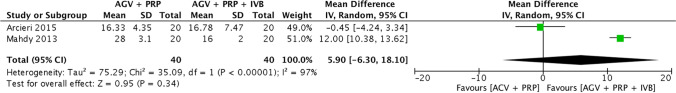
Meta-analysis of Mean IOP at 6-months follow-up. AGV, Ahmed glaucoma valve; PRP, pan-retinal photocoagulation; IVB, intra-vitreal bevacizumab; SD, standard deviation; CI, confidence interval

**Fig. 4:**
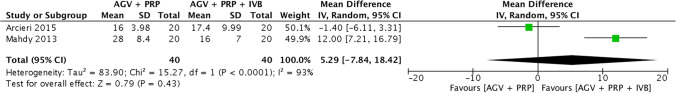
Meta-analysis of mean IOP at 12-months follow-up. AGV, Ahmed glaucoma valve; PRP, pan-retinal photocoagulation; IVB, intra-vitreal bevacizumab; SD, standard deviation; CI, confidence interval

IOP at common time points, 6 months and 12 months were analysed. There was again no statistical significance between both interventions at 6 months and 12 months (MD = 5.90, 95% CI [− 6.30, 18.10], *p* = 0.34) and (MD = 5.29, 95% CI [− 7.48, 18.42], *p* = 0.43) (Fig. 3, 4 and Table 4).

**Table 4:** Mean IOP (mmHg)

Study ID	Mean IOP ± SD (mmHg)										
	Interventions		Baseline			1 month			6 months		
	Control	Comparator	Control	Comparator	*p value*	Control	Comparator	*p value*	Control	Comparator	*p value*
AGV + PRP vs AGV + PRP + IVB											
Mahdy et al., Egypt (2013)	AGV + PRP	AGV + PRP + IVB	38.5 ± 7.5	38.4 ± 4.7	0.53	19.5 ± 2.4	13 ± 2.2	-	28 ± 3.1	16 ± 2	-
Arcieri et al., Brazil (2015)	AGV + PRP	AGV + PRP + IVB	38.35 ± 10.34	40.1 ± 13.33	0.65	19.05 ± 6.16	17.45 ± 4.65	0.359	16.33 ± 4.35	16.78 ± 7.47	0.382
Utilisation of anti-VEGF											
Yazdani et al., Iran (2009)	Sham injection	IVB	32.3 ± 14.3	33.4 ± 14.5	0.86	34.9 ± 15.2	21.8 ± 13.7	0.002	32.2 ± 7.3	23.9 ± 18.7	0.15
Wittstrom et al., Sweden (2012)	PRP	PRP + IVB	33.4 ± 17	38.1 ± 11.1	0.278	-	-	-	18.4 ± 6.8	24.8 ± 12.3	0.200
Guo et al., China (2021)	Trab + PRP + IVC	Trab + PRP + IVR	48 (41, 54)	50 (42, 54)	0.178	18 (8, 26)	19 (10, 25)	0.466	16 (10, 20)	15 (10, 23)	0.510
Inatani et al., Japan (2021)	Sham	IVA	36.7 ± 9	33 ± 10	-	-	-	-	-	-	-
Transcleral diode laser (TDL)											
Choy et al., China (2018)	TDL	AGV	42.5 ± 13.9	41.5 ± 11.5	0.9	-	-	-	-	-	-
Wagdy et al., Egypt (2020)	Express	TDL	28.2 ± 2.6	27.6 ± 4	0.532	14.48 ± 2.43	15.44 ± 2.7	0.194	-	-	-
Trabeculectomy (Trab)											
Sisto et al., Italy (2007)	Trab + MMC	Trab + 5-FU	-	-	-	-	-	-	-	-	-
Gupta et al., India (2009)	Trab + ICB 1.25	Trab + ICB 2.5	37.7 ± 15.3	33.9 ± 12.5	0.6	15.33 ± 1.6	16.4 ± 1.9	> 0.05	14 ± 4.8	11.5 ± 2	0.3
Elwehidy et al., Egypt (2019)	IVR + PRP + Trab	IVR + PRP + Vtrab	45.64 ± 3.56	45.19 ± 2.97	0.61	15.76 ± 1.16	15.46 ± 1.3	0.28	17.52 ± 0.8	16.5 ± 1.4	0.001
Tokumo et al., Japan (2021)	Baerveldt	Trab	38.9 ± 12	33.1 ± 9.3	0.1	-	-	-	11.7 ± 5.6	16.2 ± 7.5	0.03
Ahmed glaucoma valve (AGV)											
Susanna Jr et al., Brazil (2003)	AGV	High AGV + PTCR	48.45 ± 11.67	50.00 ± 10.50	> 0.05	15.19 ± 7.83	16.20 ± 10.13	> 0.05	16.08 ± 8.26	18.12 ± 8.77	> 0.05
Teixeria et al., Brazil (2012)	AGV	AGV + TCA	40.4 ± 10.8	42.1 ± 9.3	0.476	20.4 ± 9.7	13.6 ± 6.5	0.01	16.7 ± 7.7	15 ± 5.7	0.318

Study ID	Mean IOP ± SD (mmHg)									
	12 months			24 months			Final follow-up			
	Control	Comparator	*p value*	Control	Comparator	*p value*	Follow-up (months)	Control	Comparator	*p value*
AGV + PRP vs AGV + PRP + IVB										
Mahdy et al., Egypt (2013)	28 ± 8.4	16 ± 7	-	-	-	-	18	28 ± 6.5	16 ± 4.2	< 0.01
Arcieri et al., Brazil (2015)	16 ± 3.98	17.4 ± 9.99	0.459	16.67 ± 4.4	14.43 ± 0.53	0.0526	24	16.67 ± 4.4	14.43 ± 0.53	0.0526
Utilisation of anti-VEGF										
Yazdani et al., Iran (2009)	-	-	-	-	-	-	6	32.2 ± 7.3	23.9 ± 18.7	0.15
Wittstrom et al., Sweden (2012)	-	-	-	-	-	-	6	18.4 ± 6.8	24.8 ± 12.3	0.200
Guo et al., China (2021)	15 (11, 20)	16 (11, 22)	0.308	-	-	-	12	15 (11, 20)	16 (11, 22)	0.308
Inatani et al., Japan (2021)	-	-	-	-	-	-	3.25	17.6	19.8	-
Transcleral diode laser (TDL)										
Choy et al., China (2018)	-	-	-	-	-	-	TDL 28.5 ± 17.9 AGV 31.0 ± 15.4	15.2 ± 6.6	14.7 ± 4.2	0.88
Wagdy et al., Egypt (2020)	15.36 ± 1.6	15.44 ± 1.66	0.862	-	-	-	12	15.36 ± 1.6	15.44 ± 1.66	0.862
Trabeculectomy (Trab)										
Sisto et al., Italy (2007)	-	-	-	-	-	-	Trab + MMC 18.6 ± 17.2 Trab + 5-FU 35.8 ± 22.6	22.9 ± 13.3	14.7 ± 3.4	> 0.05
Gupta et al., India (2009)	-	-	-	-	-	-	6	14 ± 4.8	11.5 ± 2	0.3
Elwehidy et al., Egypt (2019)	18.76 ± 1.7	17.76 ± 2	0.02	-	-	-	12	18.76 ± 1.7	17.76 ± 2	0.02
Tokumo et al., Japan (2021)	14.3 ± 6.7	14.7 ± 4.7	0.68	13.3 ± 6.3	13.6 ± 2.5	0.9	24	13.3 ± 6.3	13.6 ± 2.5	0.9
Ahmed glaucoma valve (AGV)										
Susanna Jr et al., Brazil (2003)	18.33 ± 8.68	17.20 ± 5.05	0.51	-	-	-	12	18.33 ± 8.68	17.20 ± 5.05	0.51
Teixeria et al., Brazil (2012)	15.5 ± 4.4	13.9 ± 3.7	0.316	-	-	-	12	15.5 ± 4.4	13.9 ± 3.7	0.316

*SD*, standard deviation; *AGV*, Ahmed glaucoma valve; *High AGV*, high-pressure AGV; *PTCR*, partial Tenon’s capsule resection; *Trab*, trabeculectomy; *Vtrab*, visco-trabeculectomy; *MMC*, mitomycin C; *5-FU*, 5-flurouracil; *IVB*, intra-vitreal bevacizumab; *IVR*, intra-vitreal ranibizumab; *IVA*, intra-vitreal afilibercept; *ICB*, intra-cameral bevacizumab; *PRP*, pan-retinal photocoagulation; *TCA*, triamcinolone; *TDL*, transcleral diode laser

### Mean IOP lowering medication

Only Arcieri et al. (2015) reported mean number of IOP lowering medications at follow-up so a meta-analysis could not be carried out (Table 5). A significant difference in IOP lowering medications was only shown at 18-month follow-up, 1.67 ± 0.65 for AGV + PRP vs 1.14 ± 0.69 for AGV + PRP + IVB (*p* = 0.0002).

**Table 5:** Mean number of IOP lowering medications (mmHg)

Study ID	Mean number of IOP lowering medications ± SD										
	Interventions		Baseline			1 month			6 months		
	Control	Comparator	Control	Comparator	*p value*	Control	Comparator	*p value*	Control	Comparator	*p value*
AGV + PRP vs AGV + PRP + IVB											
Mahdy et al., Egypt (2013)	AGV + PRP	AGV + PRP + IVB	-	-	-	-	-	-	-	-	-
Arcieri et al., Brazil (2015)	AGV + PRP	AGV + PRP + IVB	2.8 ± 0.69	2.85 ± 1.18	0.391	0.7 ± 0.73	0.55 ± 0.89	0.563	1.17 ± 0.71	1.44 ± 1.25	0.511
Utilisation of anti-VEGF											
Yazdani et al., Iran (2009)	Sham injection	IVB	2.2 ± 0.7	2.0 ± 0.9	0.59	2.2 ± 0.7	1.6 + 1.1	-	2.1 ± 0.7	1.8 ± 1.2	-
Wittstrom et al., Sweden (2012)	PRP	PRP + IVB	1.7 ± 1.6	2.6 ± 1.2	0.243	-	-	-	1 ± 1.3	1.5 ± 1.4	0.423
Guo et al., China (2021)	Trab + PRP + IVC	Trab + PRP + IVR	3 (2, 4)	3 (2, 4)	0.261	2 (1, 3)	2 (1, 3)	0.720	1 (1, 2)	2 (1, 2)	0.956
Inatani et al., Japan (2021)	Sham	IVA	-	-	-	-	-	-	-	-	-
Transcleral diode laser (TDL)											
Choy et al., China (2018)	TDL	AGV	3.2 ± 0.8	2.8 ± 1.5	0.63	-	-	-	-	-	-
Wagdy et al., Egypt (2020)	Express	TDL	-	-	-	-	-	-	-	-	-
Trabeculectomy (Trab)											
Sisto et al., Italy (2007)	Trab + MMC	Trab + 5-FU	-	-	-	-	-	-	-	-	-
Gupta et al., India (2009)	Trab + ICB 1.25	Trab + ICB 2.5	-	-	-	-	-	-	-	-	-
Elwehidy et al., Egypt (2019)	IVR + PRP + Trab	IVR + PRP + Vtrab	3 ± 0	3 ± 0	-	-	-	-	-	-	-
Tokumo et al., Japan (2021)	Baerveldt	Trab	3.3 ± 1	3.6 ± 0.9	0.45	-	-	-	0.7 ± 1.1	0.8 ± 1.4	0.73
Ahmed glaucoma valve (AGV)											
Susanna Jr et al., Brazil (2003)	AGV	AGV + PTCR	-	-	-	-	-	-	-	-	-
Teixeria et al., Brazil (2012)	AGV	AGV + TCA	2.3 ± 0.9	2.4 ± 1.1	0.7	0.3 ± 0.6	0.3 ± 0.7	0.879	1 ± 1.1	0.8 ± 0.7	0.257

Study ID	Mean number of IOP lowering medications ± SD									
	12 months			24 months			Final follow-up			
	Control	Comparator	*p value*	Control	Comparator	*p value*	Follow-up (months)	Control	Comparator	*p value*
AGV + PRP vs AGV + PRP + IVB										
Mahdy et al., Egypt (2013)	-	-	-	-	-	-	-	-	-	-
Arcieri et al., Brazil (2015)	1.18 ± 0.73	1.21 ± 1.12	0.911	1.75 ± 0.62	1.14 ± 0.69	0.0648	24	1.75 ± 0.62	1.14 ± 0.69	0.0648
Utilisation of anti-VEGF										
Yazdani et al., Iran (2009)	-	-	-	-	-	-	6	2.1 ± 0.7	1.8 ± 1.2	-
Wittstrom et al., Sweden (2012)	-	-	-	-	-	-	6	1 ± 1.3	1.5 ± 1.4	0.423
Guo et al., China (2021)	2 (1, 2)	1 (1, 2)	0.772	-	-	-	12	2 (1, 2)	1 (1, 2)	0.772
Inatani et al., Japan (2021)	-	-	-	-	-	-	-	-	-	-
Transcleral diode laser (TDL)										
Choy et al., China (2018)	-	-	-	-	-	-	TDL 28.5 ± 17.9 AGV 31.0 ± 15.4	1.7 ± 1.4	0.5 ± 0.8	0.11
Wagdy et al., Egypt (2020)	-	-	-	-	-	-	-	-	-	-
Trabeculectomy (Trab)										
Sisto et al., Italy (2007)	-	-	-	-	-	-	-	-	-	-
Gupta et al., India (2009)	-	-	-	-	-	-	-	-	-	-
Elwehidy et al., Egypt (2019)	-	-	-	-	-	-	-	-	-	-
Tokumo et al., Japan (2021)	1.2 ± 1.5	1.1 ± 1.7	0.86	1.3 ± 1.6	0.6 ± 1.5	0.41	24	1.3 ± 1.6	0.6 ± 1.5	0.41
Ahmed glaucoma valve (AGV)										
Susanna Jr et al., Brazil (2003)	-	-	-	-	-	-	-	-	-	-
Teixeria et al., Brazil (2012)	1.3 ± 1.2	0.8 ± 0.8	0.284	-	-	-	12	1.3 ± 1.2	0.8 ± 0.8	0.284

*SD*, standard deviation; *AGV*, Ahmed glaucoma valve; *High AGV*, high-pressure AGV; *PTCR*, partial Tenon’s capsule resection; *Trab*, trabeculectomy; *Vtrab*, visco-trabeculectomy; *MMC*, mitomycin C; *5-FU*, 5-flurouracil; *IVB*, intra-vitreal bevacizumab; *IVR*, intra-vitreal ranibizumab; *IVA*, intra-vitreal afilibercept; *ICB*, intra-cameral bevacizumab; *PRP*, pan-retinal photocoagulation; *TCA*, triamcinolone; *TDL*, transcleral diode laser

### Complications

Throughout the follow-up periods, both studies reported complications. Mahdy et al. (2013) reported 41 complications in the AGV + PRP group and 14 in the AGV + PRP + IVB group. Of the complications reported, the ones that were common to both studies are reported here. The AGV + PRP group had 85% occurrence of hyphema, 5% of tube exposure, 10% choroidal effusion, 30% shallow/flat anterior chamber (AC), 15% hypotony and 5% phthisis bulbi. The AGV + PRP + IVB group had 20% of hyphema, 0% tube exposure, 5% choroidal effusion, 25% shallow AC, 0% phthisis bulbi and 10% hypotony (Table 6). Similarly, Arcieri et al. (2015) also reported numerically greater complications in the AGV + PRP group, 15 compared to 9 in the AGV + PRP + IVB group (Table 2). There was a 30% rate of hyphema, 20% choroidal effusion, 5% flat/shallow AC, 10% corneal oedema, 0% tube exposure and 5% retinal detachment for the AGV + PRP group. In contrast, the AGV + PRP + IVB had 10% hyphema, 15% choroidal effusion, 5% flat AC, 5% corneal oedema, 5% tube exposure and 0% retinal detachment (Table 6).

**Table 6:** All reported complications for each study

Author and year	Control	*n* number control	Comparator	*n* number comparator	Control complications *n* (%)	Comparator complications *n* (%)
AGV + PRP vs AGV + PRP + IVB						
Mahdy et al., 2013	AGV + PRP	20	AGV + PRP + IVB	20	Hyphema 17 (85), tube occlusion 1 (5), choroidal effusion 2 (10), shallow anterior chamber 6 (30), hypotony 3 (15), tube-cornea touch 2 (10), suprachoroidal haemorrhage 1 (5), phthisis bulbi 1 (5), encapsulated plate 6 (30), tube/plate exposure 1 (5), corneal decompensation 2 (10),	Hyphema 4 (20), tube occlusion 0 (0), choroidal effusion 1 (5), shallow anterior chamber 5 (25), hypotony 2 (10), tube-cornea touch 1 (5), suprachoroidal haemorrhage 0 (0), phthisis bulbi 0 (0), encapsulated plate 1 (5), tube/plate exposure 0 (0), corneal decompensation 0 (0),
Arcieri et al., 2015	AGV + PRP	20	AGV + PRP + IVB	20	Hyphema 6 (30), choroidal effusion 4 (20), flat anterior chamber 1 (5), corneal oedema 2 (10), severe inflammation 1 (5), tube exposure 0 (0), retinal detachment 1 (5)	Hyphema 2 (10), choroidal effusion 3 (15), flat anterior chamber 2 (10), corneal oedema 1 (5), severe inflammation 0 (0), tube exposure 1 (5), retinal detachment 0 (0)
Utilisation of anti-VEGF						
Yazdani et al., 2009	Sham	12	IVB	14	Not reported	Not reported
Wittstrom et al., 2012	PRP	9	PRP + IVB	10	Not reported	Not reported
Guo et al., 2021	Trab + PRP + IVC	80	Trab + PRP + IVR	80	hyphema 7 (8.75), choroidal detachment 2 (2.5), shallow anterior chamber 6 (7.5)	hyphema 9 (11.25), choroidal detachment 4 (5), shallow anterior chamber 7 (8.75)
Inatani et al., 2021	Sham	27	IVA	27	punctate keratitis 3 (11.1), eye pain 3 (11.1), conjunctival haemorrhage 1 (3.7), injection site pain 1 (3.7), procedural pain 0 (0), constipation 2 (7.4), headache 2 (7.4)	punctate keratitis 2 (7.4), eye pain 0 (0), conjunctival haemorrhage 2 (7.4), injection site pain2 (7.4), procedural pain 3 (11.1), constipation 1 (3.7), headache 1 (3.7)
Transcleral diode laser (TDL)						
Choy et al., 2018	TDL	8	AGV	13	Not reported	Intraoperative haemorrhage 1 (8), corneal decompensation 1 (8), over-filtration 2 (17), encapsulated bleb 1 (8), implant exposure 2 (17), rapid cataract progression 1 (8), phthisis bulbi 3 (25)
Wagdy et al., 2020	TDL	16	Express	12	Hyphema 1 (5.5), increase IOP 2 (11.1), hypotony 2 (11.1)	Hyphema 1 (8.3), increase IOP 2 (16.66), hypotony 3 (25)
Trabeculectomy (Trab)						
Sisto et al., 2007	Trab + MMC	22	Trab + 5FU	18	Hyphema 12 (54.5), corneal epithelial defect 0 (0), bank keratoplasty 0 (0), cataract 2 (9.1)	Hyphema 16 (88.8), corneal epithelial defects 4 (22.2), band keratoplasty 2 (11.1), cataract 0 (0)
Gupta et al., 2009	Trab + ICB 1.25	9	Trab + ICB 2.5	10	Hyphema 1 (11.1), failure 0 (0), cataract 1 (11.1), hypopyon 0 (0), posterior synechiae 3 (33)	Hyphema 0 (0), failure 1 (10), cataract 2 (20), hypopyon 1 (10), posterior synechiae 4 (40)
Elwehidy et al., 2019	IVR + PRP + Trab	25	IVR + PRP + Vtrab	26	hyphema 4 (16), filtering bleb 20 (80), encapsulated and flat bleb 5 (20), blebitis 1 (4), shallow anterior chamber 3 (12), transient hypotony 3 (12), choroidal detachment 2 (8), Descemet membrane split 0 (0), IOP spike 5 (20), progression of cataract 6 (24)	hyphema 22 (84.6), filtering bleb 3 (11.53), encapsulated and flat bleb 0 (0), blebitis 0 (0), shallow anterior chamber 1 (3.8), transient hypotony 1 (3.8), choroidal detachment 1 (3.8), Descemet membrane split 8 (30.8), IOP spike 4 (15.4), progression of cataract 2 (7.7)
Tokumo et al., 2021	Baerveldt	23	Trab	27	Hyphema 8 (34.7), choroidal detachment 6 (26.1), shallow anterior chamber 4 (17.3), tube occlusion 4 (17.3), tube exposure 5 (21.7), vitreous haemorrhage 3 (13), conjunctiva leakage 1 (4.3), expulsive haemorrhage 0 (0), endophthalmitis 0 (0)	Hyphema 9 (33.3), choroidal detachment 4 (14.8), shallow anterior chamber 2 (7.4), tube occlusion 0 (0), tube exposure 0 (0), vitreous haemorrhage 3 (11.1), conjunctiva leakage 3 (11.1), expulsive haemorrhage 1 (3.7), endophthalmitis 1 (3.7)
Ahmed glaucoma valve (AGV)						
Teixeria et al., 2012	AGV	26	AGV + TCA	23	Loss of light perception 1 (4), phthisis bulbi 1 (4), corneal decompensation 1 (4), haemorrhagic choroidal detachment 0 (0), hyphema 6 (22), hypotony 6 (22), serous choroidal detachment 3 (11), atalamy 3 (11), vitreous haemorrhage 3 (11), tube obstruction 2 (7), misdirection glaucoma 0 (0)	Loss of light perception 1 (5), phthisis bulbi 1 (5), corneal decompensation 2 (9), haemorrhagic choroidal detachment 1 (5), hyphema 4 (18), hypotony (32), serous choroidal detachment (9), atalamy (14), vitreous haemorrhage 0 (0), tube obstruction 1 (5), misdirection glaucoma 1 (5)
Susanna Jr et al., 2003	AGV	28	High AGV + PTCR	29	Retinal detachment 0 (0), hypotony 7 (25), flat anterior chamber 5 (17.9), plate exposure 1 (3.6), tube exposure 1 (3.6), tube blockage 1 (3.6), serous choroidal attachment 5 (17.9), endophthalmitis 0 (0), vitreous haemorrhage 0 (0), hyphema 8 (28.6), phthisis bulbi 0 (0)	Retinal detachment 1 (3.4), hypotony 5 (17.2), flat anterior chamber 4 (13.8), plate exposure 1 (3.4), tube exposure 2 (6.9), tube blockage 1 (3.4), serous choroidal attachment 3 (10.3), endophthalmitis 1 (3.4), vitreous haemorrhage 1 (3.4), hyphema 6 (20.7), phthisis bulbi 1 (3.4)

*AGV*, Ahmed glaucoma valve; *High AGV*, high-pressure AGV; *PTCR*, partial Tenon’s capsule resection; *Trab*, trabeculectomy; *Vtrab*, visco-trabeculectomy; *MMC*, mitomycin C; *5-FU*, 5-flurouracil; *IVB*, intra-vitreal bevacizumab; *IVR*, intra-vitreal ranibizumab; *IVA*, intra-vitreal afilibercept; *ICB*, intra-cameral bevacizumab; *PRP*, pan-retinal photocoagulation; *TCA*, triamcinolone; *TDL*, transcleral diode laser

## The utilisation of anti-VEGF therapy

### Success rates

All four studies [18–21] did not report success rates.

### Mean IOP

Inatani et al. (2021) reported mean IOP at weeks 1, 2, 5, 9 and 13 but no standard deviation or *p*-values were reported apart from week 1 where the mean IOP was 31.8 for the sham group and 24.5 for the IVA group, *p* = 0.0644. A similar study that compared sham injection vs IVB showed significantly lower IOP for IVB at 1 month (Table 4) and 3 months (35.2 ± 10.7 vs 25.1 ± 20, *p* = 0.033) [20]. Mean IOP in another study comparing PRP vs PRP + IVB showed no difference between PRP and PRP + IVB at all follow-up time points [18] (Table 4). Similarly, Guo et al. (2021) which compared Trab + PRP + IVC vs Trab + PRP + IVR did not show any significant difference between mean IOP at all follow-up time points.

### Mean IOP lowering medication

A number of IOP lowering medications were not significantly different at all follow-up times in the study by Wittstrom et al. (2012). The same is true for Guo et al. (2021). Yazdani et al. (2009) did not report statistical significance and Inatani et al. (2021) did not report the number of IOP lowering medications (Table 5).

### Complications

Wittstrom et al. (2012) and Yazdani et al. (2009) did not report complications (Table 2). Inatani et al. (2021) had a total of 20 complications in the sham injection group and 13 in the IVA group (Table 2). In the sham group, there was 11.1% punctate keratitis, 11.1% eye pain, 3.7% conjunctival haemorrhage, 3.7% injection site pain, 7.4% constipation and 7.4% headache compared with 7.4% punctate keratitis, 0% eye pain, 7.4% conjunctival haemorrhage, 7.4% injection site pain, 11.1% procedural pain, 3.7% constipation and 3.7% headache in the IVA group (Table 6). Guo et al. (2021) reported 15 complications in the Trab + PRP + IVC which consisted of 8.75% for hyphema, 2.5% for choroidal detachment and 7.5% for shallow AC. In the Trab + PRP + IVR group, there were 20 complications which consisted of 11.25% for hyphema, 5% for choroidal detachment and 8.75% for shallow AC (Tables 2, 6).

## Transcleral diode laser vs other surgical interventions

### Success rates

The definition of success was IOP < 21 mmHg with the same or improved BCVA on one study [23] and the definition of complete success was IOP < 22 mmHg and no further treatment in the second study [22]. Choy et al. (2018) showed a non-significant success rate of 63% in the TDL group and 42% in the Ahmed valve group, whereas Wagdy et al. (2020) showed a complete success rate in the TDL group compared with Express Implant group of 44.4 vs 50%, respectively. The qualified success was 41.7 vs 38.9% for Express vs TDL (Table 2).

### Mean IOP

The difference in IOP at final follow-up between TDL and the AGV was not significant [23] (Table 4). Similarly, throughout the 12-month follow-up in the study by Wagdy et al. (2020), there was no significant difference between IOP at all follow-up time points.

### Mean IOP lowering medications

Wagdy et al. (2020) did not report results on IOP lowering medication whereas Choy et al. (2018) did and showed a non-significant difference in IOP lowering medications at final follow-up between TDL and the AGV (Table 5).

### Complications

Regarding complications, Choy et al. (2018) only reported complications for the AGV group which was a total of 11. These complications comprised 8% intraoperative haemorrhage, 8% corneal decompensation, 17% over-filtration, 8% encapsulated bleb, 17% implant exposure, 8% rapid cataract progression and 25% phthisis bulbi (Table 6). In the other study, the TDL group had a total of 5 complications and the Express Implant group had a total of 6 [22]. The TDL group reported 5.5% of hyphema, 11.1% increased IOP and 11.1% hypotony whereas the Express group reported 8.3% for hyphema, 16.66% for increased IOP and 25% for hypotony (Table 6).

## Trabeculectomy

### Success rates

There was a complete success rate, defined as IOP < 21 mmHg with no further IOP lowering medications or surgeries, of 52% in the IVR + PRP + Trab group and 53.8% in the IVR + PRP + VTrab [25]. Qualified success in this study was 28% for the Trab group and 30.8% in the Vtrab group and defined as IOP 6–21 mmHg with IOP lowering medications. Neither was significant [25]. In another study, Trab had a success rate, defined as IOP < 22 mmHg with no further surgeries and no decrease in BCVA, of 61.6%, and the Baerveldt group had a success rate of 59.1% which were also not significantly different [24] (Table 2). When comparing Trab + MMC with Trab + 5-FU, there was an overall success rate, IOP > 21 mmHg with or without topical treatment, of 54.5% for the MMC group vs 55.5% for the 5-FU group [26]. The study by Gupta et al. (2009) did not report success rates.

### Mean IOP

During all follow-up times, there was a significantly lower mean IOP at 6 months (Table 4), 9 months (18 ± 1.5 vs 17.19 ± 1.6 mmHg), 12 months (Table 4) and 18 months (18.19 ± 2.0 vs 19.92 ± 2.6) for the Vtrab group [25]. The study by Tokumo et al. (2021) showed a significance in mean post-operative IOP at 6 months only for the Baerveldt group (Table 4). Gupta et al. (2009) and Sisto et al. (2007) did not report a difference in the IOP at all follow-up time points (Table 4).

### Mean IOP lowering medication

There was no difference in the mean number of IOP lowering medications between the Baerveldt and Trab groups [24]. However, Elwehidy et al. (2018), Gupta et al. (2009) and Sisto et al. (2007) did not report results on IOP lowering medication (Table 5).

### Complications

There were a total of 31 complications in the Baerveldt group and 23 in the trabeculectomy group [24]. In the study by Tokumo et al. (2021), there was a total of 49 complications in the Baerveldt group which consisted of 34.7% hyphema, 26.1% choroidal detachment, 17.3% shallow AC, 17.3% tube occlusion, 13% vitreous haemorrhage, 4.3% conjunctival leakage and 0% endophthalmitis. In the Trab group, there were 42 complications which consisted of 33.3% hyphema, 14.8% choroidal detachment, 7.4% shallow AC, 11.1% vitreous haemorrhage, 11.1% conjunctival leakage and 3.7% endophthalmitis (Table 6). In the Elwehidy study, they were 84.6% for hyphema, 3.8% shallow AC, 3.8% hypotony, 3.8% choroidal detachment, 30.76% for Descemet membrane split, 15.38% IOP spike and 7.7% for progression of cataract in the Vtrab group and 16% for hyphema, 80% filtering bleb, 20% encapsulated bleb, 4% blebitis, 12% shallow AC, 12% hypotony, 8% choroidal detachment, 20% IOP spikes and 24% for progression of cataract in the Trab group (Table 6).

Gupta et al. (2009) reported 5 (11% hyphema, 0% failure, cataract 11%, hypopyon 0% and 33% for posterior synechiae) and 8 complications (0% hyphema, 10% for failure, 20% cataract, 10% hypopyon and 40% for posterior synechiae) for Trab + ICB 1.25 and Trab + ICB 2.5, respectively. Sisto et al. (2007) reported 14 complications in the Trab + MMC group, 54.5% hyphema and 9.1% cataract, and 22 in the Trab + 5-FU group, 88.8% hyphema, 22.2% corneal epithelial defects and 11.1% band keratoplasty (Tables 2 and 6).

## AGV vs other interventions

### Success rates

For AGV vs AGV + TCA, the complete and overall success rates were not significantly different. The AGV group had a complete success rate of 64 vs 77.8% and an overall success rate of 76 vs 77.8% [28]. The definition for complete success in this study was IOP < 21 mmHg and > 6 mmHg using < 2 IOP lowering medications and overall success was the same definition but any number of IOP lowering medications (Table 2). Susanna Jr et al. (2003) defined success as IOP between 4 and 22 mmHg and at least 30% reduction in IOP ± IOP lowering medication. Using this definition, 77.7% of the patients in the AGV group had a successful procedure compared to 70.4% in the High AGV + PTCR which was deemed not significant (Table 2).

### Mean IOP

Only at the 1-month follow-up was there a significant lower mean IOP post-operatively in the AGV + TCA group [28] (Table 4). Whereas for the study carried out by Susanna Jr et al. (2003), there was no significant difference in the mean IOP at all follow-up time points (Table 4.)

### Mean IOP lowering medications

There was no difference in the mean number of IOP medications at any follow-up time point [28] and Susanna Jr et al. (2003) did not report the mean number of IOP lowering medications (Table 5).

### Complications

The total number of complications in the AGV group was 26 [28]. They involved 4% loss of light perception, 4% phthisis bulbi, 4% corneal decompensation, 22% hyphema, 22% hypotony, 11% serous choroidal detachment, 11% vitreous haemorrhage and 7% tube obstruction. For AGV + TCA, there were 23 complications which consisted of 5% loss of light perception, 5% phthisis bulbi, 9% corneal decompensation, 5% haemorrhagic choroidal detachment, 18% hyphema, 32% hypotony, 9% serous choroidal detachment, 0% vitreous haemorrhage and 5% tube obstruction (Table 6).

In the other study [29], there were 28 reported complications in the AGV group (0% retinal detachment, 25% hypotony, 17.9% flat AC, 3.6% tube exposure, 3.6% plate exposure, 3.6% tube blockage, 17.9% serous choroidal detachment, 0% endophthalmitis or vitreous haemorrhage, 28.6% hyphema and no phthisis bulbi). In contrast, there was 29 (3.4% retinal detachments, 17.2% hypotony, 13.8% flat AC, 6.9% tube exposure, 3.4% plate exposure, 3.4% tube blockage, 10.3% serous choroidal detachment, 3.4% endophthalmitis, 3.4% vitreous haemorrhage, 20.7% hyphema, 3.4% phthisis bulbi) in the High AGV + PTCR group (Table 2 and 6).

### GRADE analysis

GRADE analysis was carried out for each of the 4 outcomes. Results are presented in Table 7. Overall GRADE rating was low for success rate and moderate for mean IOP, mean number of IOP lowering medications and total complications.

**Table 7:** Quality of evidence of each outcome as assessed by the GRADE system

Outcomes		No. of studies	Risk of bias	Imprecision	Inconsistency	Indirectness	Publication bias	Overall GRADE rating
Primary	Mean IOP	14	Moderate	Low	Low	Low	Low	Moderate
	Success rate	9	High	Low	High	Low	Low	Low
Secondary	Mean IOP lowering medications	7	Moderate	Low	Low	Low	Low	Moderate
	Total complications	13	Moderate	Low	Low	Low	Low	Moderate

## Discussion

### Summary of results

In the past 21 years, only 2 RCTs comparing treatments for NVG were eligible for meta-analysis. They showed no mean difference between IOP at 6 and 12 months as well as similar odds for success between AGV + PRP and AGV + PRP + IVB [16, 17]. Regarding anti-VEGF and its utilisation, one study from four showed lower mean IOP at 1 (*p* = 0.002) and 3 months (*p* = 0.33) for IVB vs sham injection [20]. From the four studies investigating trab, lower mean IOP was present for IVR + PRP + Vtrab vs IVR + PRP + trab at at 6 (*p* = 0.001), 9 (*p* = 0.01), 12 (*p* = 0.02) and 18 months (*p* = 0.004) [25]. Also, there was lower mean IOP at 6 months (*p* = 0.03) for the Baerveldt group vs trab [24]. In the two studies investigating AGV, there was a lower mean IOP at 1 month (*p* = 0.01) in the AGV + TCA group [28]. For the two papers studying TDL, neither had significant results.

### Previous systematic reviews and meta-analyses

Previous systematic reviews have used data from prospective and retrospective cohort studies as there are few RCTs on the management of this disease. One previous systematic review evaluated the surgical treatment of NVG with 7 non-randomised studies [7]. Schomak and colleagues compared glaucoma drainage devices (GDDs) with cyclophotocoagulation (also known as TDL) as well as AGV with trabeculectomy. They showed that GDDs and cyclophotocoagulation had similar IOP lowering efficacy (WMD =  − 3.63; CI [− 8.69, 1.43]; *p* = 0.16) but cyclophotocoagulation had greater rates of failure and loss of light perception (RR = 0.64, CI [0.41, 0.99], *p* = 0.05 & (RD =  − 0.15, CI [− 0.25, − 0.05], *p* = 0.004) [7]. In our review, two studies compared GDDs, AGV [23] and Express [22], with TDL. Just as the meta-analysis showed similar IOP lowering efficacy, both of these studies did not show a difference in mean IOP at follow-up, but there was a greater percentage of success for TDL vs AGV [23]. Schomak et al. (2019) also stated that the AGV had greater IOP lowering efficacy compared with Trab (WMD = 0.78; CI [− 2.29, 3.85], *p* = 0.62) but a higher failure rate (RR = 2.25, CI [1.14, 3.71], *p* = 0.02).

Two previous systematic reviews have also been conducted that looked at the clinical outcomes of AGV with or without IVB [30, 31]. Zhou et al. (2015) concluded that there was a similar IOP lowering efficacy between AGV and AGV + IVB (WMD = 3.30; 95% CI 1.21 to 7.80, *p* = 0.152), a greater complete success rate in the AGV + IVB group and that the reduction in IOP lowering medications was similar between both groups (WMD = 0.28; 95% CI − 0.03 to 0.59, *p* = 0.077). The second systematic review showed similar results in that success rate was greater in the AGV + IVB group and that in this group, the incidence of complications such as hyphema, vitreous haemorrhage and hypotony was lower [31]. Both reviews included non-randomised studies as well as RCTs. Comparing these results to our meta-analysis, the RCTs show no mean difference between IOP at 6 and 12 months as well as similar odds for success between AGV + PRP and AGV + PRP + IVB [16, 17]. Furthermore, only at 18 months was there a lower number of IOP lowering medications for the AGV + PRP + IVB group [17]. The difference between our meta-analysis and those carried out by Zhou et al. (2015) and Hwang et al. (2015) is that we only included RCTs whilst both other authors included non-randomised studies as well. This highlights the need for more RCTs investigating treatments for NVG as they generate a higher quality of evidence.

With other systematic reviews’ results showing that the addition of anti-VEGF medications can improve clinical outcomes in patients with NVG [30, 31], this poses the question are they beneficial only as adjuncts or as sole interventions. A Cochrane review in 2020 [32] identified 4 studies, of which 1 was an ongoing study, evaluating anti-VEGF for the treatment of NVG. Three of those studies [17, 19, 22] have been included in this review; however, Jiang et al. (2015) was not as there was no English translation at the time of search and the article was published in Mandarin. Our review identified 2 studies that compared sham injection with anti-VEGF, IVB [20] or IVA [19]. However, a meta-analysis could not be carried out due to both not reporting success rates and no standard deviations being reported by Inatani et al. (2021). The results showed that Yazdani et al. (2009) reported lower mean IOP for IVB vs sham injection at 1 and 3 months but a non-significant IOP at 6 months. On the other hand, Inatani et al. (2021) did not report *p*-values and therefore conclusions regarding the IOP lowering efficacy of IVA could not be made. The results suggest that anti-VEGF does provide significant IOP lowering in neovascular glaucoma, but similar to our situation where a meta-analysis could not be conducted, the Cochrane review also identified that a meta-analysis could not be carried out because there were very few RCTs with large heterogeneity between them; instead, a narrative synthesis was done, similar to our review.

### A review of literature

Both the AGV and Baerveldt implant have been discussed in this review. However, in recent years, there have been large pivotal randomised studies that have investigated the use of these implants over a longer time period. Two of these studies are the AGV Baerveldt Comparison (ABC) study [33] and the AGV vs Baerveldt (AVB) study [34]. The ABC study demonstrated that the Baerveldt was more effective in long-term IOP control but had similar rates of failure to the AGV. There was a subset of 57 NVG patients of which 29 were allocated to AGV and 28 to the Baerveldt group. For both groups, failure and success rates were reported. The failure was 66%, complete success 3% and qualified success 31% for the AGV group and 71%, 21% and 7% for the Baerveldt group, respectively. The AVB study reviewed 5-year treatment outcomes and concluded lower mean IOP and glaucoma medications in the Baerveldt group, higher failure in AGV group and similar visual acuity and success rates between both groups. Two hundred and thirty-eight patients were enrolled in this study. Fifty, 21%, of participants enrolled had neovascular glaucoma with 28 allocated to the AGV group and 22 to the Baerveldt group. However, different to the ABC study, there was no sub-group analysis carried out. Although the ABC study had some sub group analysis for success and failure, the sub group analysis of other outcomes is missing. Our inclusion criteria were studies investigating NVG patients only and the purpose of these studies was not to investigate the management of NVG. A suggestion for future reviews could be to investigate all glaucoma studies and try tease out data from NVG sub-group analyses, but with the vast number of trials in the literature, this would be extremely time-consuming for the amount of data available.

### Limitations

Overall, the main limitation is the significant paucity of well-constructed RCTs in the management of this disease. Current practice seems to be based on case series, expert opinions and non-randomised studies rather than well-designed clinical trials. Similarly, anti-VEGF treatments are also widely used for the treatment of NVG but Simha et al. (2020) was unable to draw definitive conclusions from their review of RCTs. In addition, there is a need for studies to have longer-term follow-ups. Many studies report short-term outcomes but long-term IOP control is necessary as glaucoma is a life-long disease. This has not only been identified in this systematic review but previous reviews too.

Furthermore, there is the limitation of publication bias in our study as well as heterogeneity between studies in the criteria for success. The majority of studies in our review accepted success as IOP < 21–22 mmHg and/or no further interventions to reduce IOP, but as can be seen in Table 2, the definition of success varied between studies. The heterogeneity highlighted here was present between the studies included in the meta-analysis. In order to deal with this, a standard outcome measure of complete success was utilised as it was used by most RCTs. Additionally, a random effects model was used for meta-analysis and appropriate statistical analysis, *I*^2^, was used which reported results of 77%, 97% and 93% for outcome measures for AGV + PRP vs AGV + PRP + IVB, respectively. However, given the limitations for the majority of trials, only a narrative synthesis was completed.

### Recommendations

To truly appreciate relationships, efficacy and safety, a universal NVG core outcome set is required. This should clearly define success and failure alongside provide reporting guidance on vital outcomes (e.g. mean IOP/mean IOP reduction and mean number of IOP lowering medications), common complications (e.g. hyphema, tube exposure or even hypotony) and baseline ocular demographic details. Standardised follow-up time-points that include long-term follow-up (1 week, 1, 3, 6, 12, 24 months at a minimum) should be encouraged. A unison method of reporting will support in determining true treatment effect sizes in the future.

### Agreement

Our results suggest an absence of consensus and variability of evidence, which is likely the cause for variance in practice seen between ophthalmologists when treating this disease. We believe a combination of VEGF suppression and shunt-based procedure would be beneficial to those requiring surgical intervention, with cyclodiode therapy used as an alternative adjunct—showing good IOP reduction (not without its own risks and sight threatening complications). More recent literature on the utilisation of surgical micro-shunts in the treatment of secondary glaucoma has shown promising results [35]. Further work must be done to look at the potential role of interventional glaucoma and long-term outcomes in patients with neovascular glaucoma. Finally, additional work must be done to determine the most suitable time for implementation of medical therapy such as anti-VEGF; this can potentially be done with the utilisation of information gained through omics work done on vitreous concentration studies. This information can be hypothetically used to risk stratify individuals whilst identifying those with the most aggressive forms of disease and likely poor outcomes.

## Conclusion

In conclusion, from our search, only 14 RCTs were published in the last 2 decades and the majority of them compared different treatments. Not all studies reported the desired outcomes of mean IOP lowering medications and success rates. Due to the large heterogeneity between studies, a meta-analysis could only be carried out between 2 studies and a narrative synthesis had to be carried out for the remaining 12 studies. This significant heterogeneity in methods highlights that there is still no established optimal medical or surgical treatment of choice as well as processes of risk stratifying patients. Our results highlight that there is a great need for RCTs with large numbers of participants and extensive follow-up in order to meta-analyse data and clarify the best treatments for NVG. Ideally, a core outcome set is needed so that trials can then be combined and a meta-analysis performed. Moreover, studies should group together patients with the same underlying aetiology as this may modify the effectiveness of treatment.

## Supplementary Information

Below is the link to the electronic supplementary material.

Supplementary file1 (DOCX 69 KB)

Supplementary file2 (PDF 478 KB)

## References

[1] Shazly TA, Latina MA (2009). Neovascular glaucoma: etiology, diagnosis and prognosis. Semin Ophthalmol.

[2] Hayreh SS (2007). Neovascular glaucoma. Prog Retin Eye Res.

[3] Aiello LP, Avery RL, Arrigg PG, Keyt BA, Jampel HD, Shah ST, Pasquale LR, Thieme H, Iwamoto MA, Park JE (1994). Vascular endothelial growth factor in ocular fluid of patients with diabetic retinopathy and other retinal disorders. N Engl J Med.

[4] Tolentino MJ, Miller JW, Gragoudas ES, Chatzistefanou K, Ferrara N, Adamis AP (1996). Vascular endothelial growth factor is sufficient to produce iris neovascularization and neovascular glaucoma in a nonhuman primate. Arch Ophthalmol.

[5] Chalam KV, Brar VS, Murthy RK (2014). Human ciliary epithelium as a source of synthesis and secretion of vascular endothelial growth factor in neovascular glaucoma. JAMA Ophthalmol.

[6] Sivak-Callcott JA, O’Day DM, Gass JD, Tsai JC (2001). Evidence-based recommendations for the diagnosis and treatment of neovascular glaucoma. Ophthalmology.

[7] Shchomak Z, Cordeiro Sousa D, Leal I, Abegão Pinto L (2019). Surgical treatment of neovascular glaucoma: a systematic review and meta-analysis. Graefes Arch Clin Exp Ophthalmol.

[8] Parkash D, Gohil A, Ansari AS, Kulkarni A, Kailani O (2022). Trends in glaucoma surgery: an evolving landscape. Acta Ophthalmol.

[9] Hernandez-Oteyza A, Lazcano-Gomez G, Jimenez-Roman J, Hernandez-Garciadiego C (2014). Surgical outcome of ahmed valve implantation in mexican patients with neovascular glaucoma. J Curr Glaucoma Pract.

[10] Mietz H, Raschka B, Krieglstein GK (1999). Risk factors for failures of trabeculectomies performed without antimetabolites. Br J Ophthalmol.

[11] Olmos LC, Lee RK (2011). Medical and surgical treatment of neovascular glaucoma. Int Ophthalmol Clin.

[12] Lee E, Ansari AS, Nagi G, Ramji S, Jackson T, Kailani O (2022). Evaluating variance in the medical and surgical management of neovascular glaucoma. Acta Ophthalmol.

[13] Ouzzani M, Hammady H, Fedorowicz Z, Elmagarmid A (2016). Rayyan — a web and mobile app for systematic reviews. Syst Rev.

[14] Sterne JAC, Savović J, Page MJ (2019). RoB 2: a revised tool for assessing risk of bias in randomised trials. BMJ.

[15] Guyatt GH, Oxman AD, Vist GE, Kunz R, Falck-Ytter Y, Alonso-Coello P, Schünemann HJ (2008). GRADE: an emerging consensus on rating quality of evidence and strength of recommendations. BMJ.

[16] Mahdy RA, Nada WM, Fawzy KM, Alnashar HY, Almosalamy SM (2013). Efficacy of intravitreal bevacizumab with panretinal photocoagulation followed by Ahmed valve implantation in neovascular glaucoma. J Glaucoma.

[17] Arcieri ES, Paula JS, Jorge R, Barella KA, Arcieri RS, Secches DJ, Costa VP (2015). Efficacy and safety of intravitreal bevacizumab in eyes with neovascular glaucoma undergoing Ahmed glaucoma valve implantation: 2-year follow-up. Acta Ophthalmol.

[18] Wittström E, Holmberg H, Hvarfner C, Andréasson S (2012). Clinical and electrophysiologic outcome in patients with neovascular glaucoma treated with and without bevacizumab. Eur J Ophthalmol.

[19] Inatani M, Higashide T, Matsushita K, Miki A, Ueki M, Iwamoto Y, Kobayashi M, Leal S, Investigators VEGA (2021). Intravitreal aflibercept in Japanese patients with neovascular glaucoma: the VEGA randomized clinical trial. Adv Ther.

[20] Yazdani S, Hendi K, Pakravan M, Mahdavi M, Yaseri M (2009). Intravitreal bevacizumab for neovascular glaucoma: a randomized controlled trial. J Glaucoma.

[21] Guo X, Wang Y, Yang L, Wang P, Chen K, Zhou L, Wu Y (2021). Comparison of conbercept and ranibizumab combined mitomycin C-augmented trabeculectomy for neovascular glaucoma. Int Ohthalmol.

[22] Wagdy FM, Zaky AG (2020). Comparison between the express implant and transscleral diode laser in neovascular glaucoma. J Ophthalmol.

[23] Choy B, Lai J, Yeung J, Chan J (2018). Randomized comparative trial of diode laser transscleral cyclophotocoagulation versus Ahmed glaucoma valve for neovascular glaucoma in Chinese - a pilot study. Clin Ophthalmol.

[24] Tokumo K, Komatsu K, Yuasa Y, Murakami Y, Okumichi H, Hirooka K, Nakakura S, Tabuchi H, Kiuchi Y (2021). Treatment outcomes in the neovascular glaucoma tube versus trabeculectomy study. Graefes Arch Clin Exp Ophthalmol.

[25] Elwehidy AS, Bayoumi N, Badawi AE, Hagras SM, Abdelkader A (2019). Intravitreal ranibizumab with panretinal photocoagulation followed by trabeculectomy versus visco-trabeculotomy in management of neovascular glaucoma. Asia Pac J Ophthalmol (Phila).

[26] Sisto D, Vetrugno M, Trabucco T, Cantatore F, Ruggeri G, Sborgia C (2007). The role of antimetabolites in filtration surgery for neovascular glaucoma: intermediate-term follow-up. Acta Ophthalmol Scand.

[27] Gupta V, Jha R, Rao A, Kong G, Sihota R (2009). The effect of different doses of intracameral bevacizumab on surgical outcomes of trabeculectomy for neovascular glaucoma. Eur J Ophthalmol.

[28] Teixeira SH, Doi LM, Freitas Silva AL, Silva KD, Paes AT, Higa FS, Mendonça M, Prata JA, Paranhos A (2012). Silicone Ahmed glaucoma valve with and without intravitreal triamcinolone acetonide for neovascular glaucoma: randomized clinical trial. J Glaucoma.

[29] Susanna R, Latin American Glaucoma Society Investigators (2003). Partial Tenon’s capsule resection with adjunctive mitomycin C in Ahmed glaucoma valve implant surgery. Br J Ophthalmol.

[30] Zhou M, Xu X, Zhang X, Sun X (2016). Clinical outcomes of ahmed glaucoma valve implantation with or without intravitreal bevacizumab pretreatment for neovascular glaucoma: a systematic review and meta-analysis. J Glaucoma.

[31] Hwang HB, Han JW, Yim HB, Lee NY (2015). Beneficial effects of adjuvant intravitreal bevacizumab injection on outcomes of Ahmed glaucoma valve implantation in patients with neovascular glaucoma: systematic literature review. J Ocul Pharmacol Ther.

[32] Simha A, Aziz K, Braganza A, Abraham L, Samuel P, Lindsley KB (2020) Anti‐vascular endothelial growth factor for neovascular glaucoma. Cochrane Database of Systematic Reviews, Issue 2, Art. No: CD007920. 10.1002/14651858.CD007920.pub3 10.1002/14651858.CD007920.pub3 PMC7003996 32027392

[33] Budenz DL, Barton K, Gedde SJ, Feuer WJ, Schiffman J, Costa VP, Godfrey DG, Buys YM, The Ahmed Baerveldt Comparison Study Group (2015). Five-year treatment outcomes in the Ahmed Baerveldt comparison study. Ophthalmology.

[34] Christakis PG, Kalenak JW, Tsai JC, Zurakowski D, Kammer JA, Harasymowycz PJ, Mura JJ, Cantor LB, Ahmed IIK (2016). The Ahmed versus baerveldt study: five-year treatment outcomes. Ophthalmology.

[35] Ibarz Barbera M, Morales-Fernandez L, Gómez de Liaño R, Tañá Rivero P, Teus MA (2021). Changes to corneal topography and biometrics after PRESERFLO microshunt surgery for glaucoma. J Glaucoma.

