# Supplementary material for: A systematic review and meta-analysis of randomised controlled trials in the management of neovascular glaucoma: absence of consensus and variability in practice
Source: Graefes Arch Clin Exp Ophthalmol. 2022 Aug 8;261(2):477–501. doi: 10.1007/s00417-022-05785-5 (PMC9358103; doi:10.1007/s00417-022-05785-5)
Supplement: Supplementary file 1 — Supplementary file1 (DOCX 69 KB) [file 417_2022_5785_MOESM1_ESM.docx]

**Appendix A**

**Example of search strategy**

1.) Iris rubeosis.mp.

2.) Neovascular glaucoma.mp.

3.) NVG.mp.

4.) Rubeosis iridis.mp.

5.) Rubeotic glaucoma.mp.

6.) Iris neovascular*.mp

7.) anterior neovascular*.mp

8.) neovascular* of iris.mp

9.) neovascular* of angle.mp

10.) NVI.mp

11.) angle rubeosis.mp

12.) laser.mp

13.) photocoagulation.mp

14.) PRP.mp

15.) panretinal photocoagulation.mp

16.) transceral photocoagulation.mp

17.) endophotocoagulation.mp

18.) Transcleral cyclophotocoagulation.mp

19.) endocyclophotocoagulation.mp

20.) Anti-VEGF.mp

21.) anti-vascular endothelial growth factor.mp

22.) Aflibercept.mp

23.) Eylea.mp

24.) Zaltrap.mp

25.) Bavacizumab.mp

26.) Avastin

27.) Altzan.mp

28.) Ranibizumab.mp

29.) Lucentis.mp

30.) Rhufab.mp

31.) Pegatanib.mp

32.) Macugen.mp

33.) Angiostatic.mp

34.) anti-angiogenic.mp

35.) anti-angiogenetic.mp

36.) anti-angiogensis.mp

37.) antiangiogenic.mp

38.) antiangiogenetic.mp

39.) antiangiogensis.mp

40.) Surg*.mp

41.) Glaucoma drainage device*.mp

42.) drain*.mp

43.) Molteno.mp

44.) Baerveldt.mp

45.) Ahmed glaucoma valve.mp

46.) Ahmed valve.mp

47.) Shunt*.mp

48.) tube*.mp

49.) Filtering surg*.mp

50.) Trabeculect*.mp

51.) Cyclodestruct*.mp

52.) Cryotherapy.mp

53.) Cycloplast*.mp

54.) eye drop*.mp

55.) b-adrenergic antagonist*.mp

56.) beta adrenergic antagonist*.mp

57.) beta blocker*.mp

58.) b-blocker*.mp

59.) beta antagonist*.mp

60.) b-antagonist*.mp

61.) betaxolol.mp

62.) levobunolol.mp

63.) timolol.mp

64.) alpha-2 agonist*.mp

65.) a-2 agonist*.mp

66.) alpha-2 adrenergic agonist*.mp.

67.) a-2 adrenergic agonist*.mp

68.) alpha agonist*.mp.

69.) a-agonist*.mp

70.) a-adrenergic agonist*.mp.

71.) alpha adrenergic agonist*.mp

72.) Brimonidine.mp

73.) Apraclonidine.mp

74.) Alphagan.mp

75.) Iopidine.mp

76.) Carbonic anhydrase.mp

77.) acetazolamide.mp

78.) methazolamide.mp

79.) dorzolamide.mp

80.) Trusopt.mp

81.) Azopt.mp

82.) Diamox.mp

83.) Neptazane.mp

84.) Prostaglandin analog*.mp

85.) latonoprost.mp

86.) bimatoprost.mp

87.) tavoprost.mp

88.) tafluprost.mp

89.) latanoprostene bunod.mp

90.) Xalatan.mp

91.) Lumigan.mp

92.) Travatan.mp

93.) Zioptan.mp

94.) Vyzulta.mp

95.) 1 or 2 or 3 or 4 or 5 or 6 or 7 or 8 or 9 or 10 or 11

96.) 12 or 13 or 14 or 15 or 16 or 17 or 18 or 19

97.) 20 or 21 or 22 or 23 or 24 or 25 or 26 or 27 or 28 or 29 or 30 or 31 or 32 or 33 or 34 or 35 or 36 or 37 or 38 or 39

98.) 40 or 41 or 42 or 43 or 44 or 45 or 46 or 47 or 48 or 49 or 50 or 51 or 52 or 53

99.) 54 or 55 or 56 or 57 or 58 or 59 or 60 or 61 or 62 or 63 or 64 or 65 or 66 or 67 or 68 or 69 or 70 or 71 or 72 or 73 or 74 or 75 or 76 or 77 or 78 or 79 or 80 or 81 or 82 or 83 or 84 or 85 or 86 or 87 or 88 or 89 or 90 or 91 or 92 or 93 or 94

100.) 96 or or 97 or 98 or 99

101.) 95 and 100

102.) Removal of duplicates from 101

103.) 102 and 2000:2021.(sa_year)
